# Time-Resolved Spectroscopic and Density Functional Theory Investigation of the Photogeneration of a Bifunctional Quinone Methide in Neutral and Basic Aqueous Solutions

**DOI:** 10.3390/molecules23123102

**Published:** 2018-11-27

**Authors:** Zhiping Yan, Lili Du, Xin Lan, Yuanchun Li, Wenchao Wang, David Lee Phillips

**Affiliations:** Department of Chemistry, The University of Hong Kong, Hong Kong, China; mcayzp@gmail.com (Z.Y.); xinlan@connect.hku.hk (X.L.); fionalyc@connect.hku.hk (Y.L.); wenchao0909@126.com (W.W.)

**Keywords:** bifunctional quinone methides, time-resolved resonance Raman, DFT calculation, E1bc elimination reaction

## Abstract

Binol quinone methides (BQMs) can be generated from 1,1′-(2,2′-dihydroxy-1,1′-binaphthyl-6,6′-diyl)bis(*N*,*N*,*N*-trimethylmethanamiuium) bromide (BQMP-b) in a 1:1 MeCN:H_2_O mixed solution via a ground state intramolecular proton transfer (GSIPT), as mentioned in our previously reported studies. Here, the photoreaction of BQMP-b in neutral and basic aqueous solution (pH = 7, 10, 12) was investigated to explore the possible mechanisms and the key intermediates produced in the process of the photoreaction and to examine whether they are different from those in a neutral mild-mixed MeCN:H_2_O solution. The studies were conducted using femtosecond transient absorption (fs-TA), nanosecond transient absorption (ns-TA), and nanosecond time-resolved resonance Raman spectroscopy (ns-TR^3^) in conjunction with results from density functional theory (DFT) computations. The results showed that BQMP-b was deprotonated initially and produced BQMs species more effectively through an E1bc elimination reaction in a strong basic aqueous condition (pH = 12), which differed from the reaction pathway that took place in the solution with pH = 7 or 10. A related single naphthol ring molecule 1-(6-hydroxynaphthalen-2-yl)-*N*,*N*,*N*-trimethylmethanaminium bromide (QMP-b) that did not contain a second naphthol ring was also investigated. The related reaction mechanisms are elucidated in this work, and it is briefly discussed how the mechanisms vary as a function of aqueous solution pH conditions.

## 1. Introduction

**Scheme 1:**
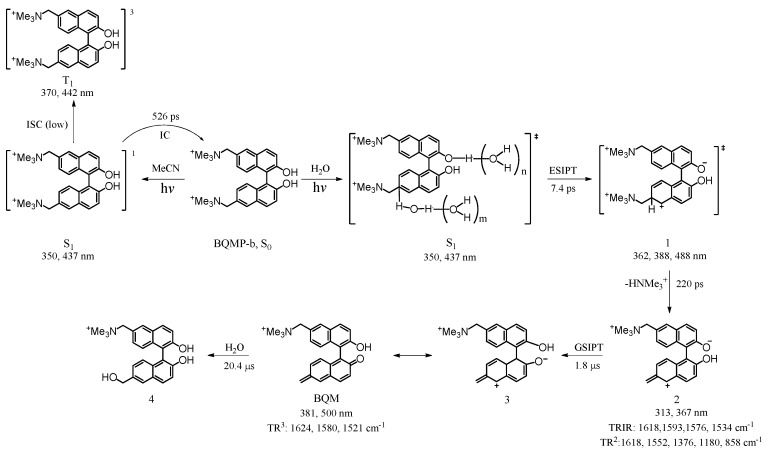
Mechanism proposed by Du and co-workers for the water-assisted photoreaction of 1,1′-(2,2′-dihydroxy-1,1′-binaphthyl-6,6′-diyl)bis(*N*,*N*,*N*-trimethylmethanamiuium) bromide (BQMP-b) [17], the (‡) marks the excited state.

In recent years, bifunctional quinone methides (QMs) have attracted more attention from scientists because they have been discovered to be important intermediates to alkylate and crosslink DNA, thus giving them the potential to be applied in cancer therapy [1,2,3,4,5,6]. Bifunctional QMs can be generated photochemically from suitably bifunctional substituted naphthol derivatives, such as the corresponding Mannich bases and their ammonium salts and hydroxybenzyl alcohols and their methyl ethers [7,8,9,10,11,12,13,14]. Freccero et al. reported on the chemical and spectroscopic behavior of binol quaternary ammonium derivatives and studied their bis-alkylation processes in water and DNA interstrand cross-linking reactions. They pointed out that the reactivity of binol quinone methides (BQMs) generated from the derivatives was very stable and that they were useful DNA cross-linkers with submicromolar potency using UV-visible light activation [15,16]. In light of the importance of BQMs, the elucidation of the mechanism of their formation is important to better understand how BQMs are formed and to determine their yields under varying conditions. In our previous work, the transient species BQMs were found to be generated in the photoexcitation of 1,1′-(2,2′-dihydroxy-1,1′-binaphthyl-6,6′-diyl)bis(*N*,*N*,*N*-trimethylmethanamiuium) bromide (BQMP-b) in which a water-assisted excited-state intramolecular proton transfer (ESIPT) first occurred and then the BQM species was produced via a ground state intramolecular proton transfer (GSIPT) reaction [17,18]. The proposed mechanism for this kind of pathway is displayed in Scheme 1.

Although many meaningful results have been obtained from previous research studies, the photoreactions of interest mainly occurred under mild conditions, and the excited-state proton transfer (ESPT) or ESIPT processes were typically suggested to take place in all of these systems [19,20]. To the best of our knowledge, few investigations have been done to study the same photophysical and photochemical events that occur under alkaline aqueous solutions, and some vital questions remain unanswered. For example, is there any possible mechanism except ESIPT or ESPT for the charge transfer under alkaline aqueous systems? Are BQMs still formed in the photoreaction of BQMP-b when OH^−^ abounds in the solution? If yes, how are the reactive BQMs produced and what is the effect on the yield of the BQMs? More experimental study is necessary to further elucidate the details of the reactive intermediates and mechanisms for the photoreactions under these alkaline aqueous conditions.

**Scheme 2:**
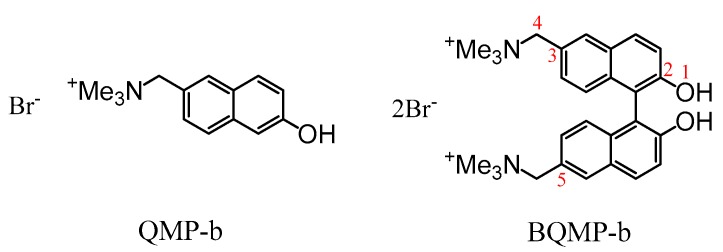
The structures of 1-(6-hydroxynaphthalen-2-yl)-*N*,*N*,*N*-trimethylmethanaminium bromide (QMP-b) and 1,1′-(2,2′-dihydroxy-1,1′-binaphthyl-6,6′-diyl)bis(*N*,*N*,*N*-trimethylmethanamiuium) bromide (BQMP-b).

In this work, we conducted a combined nanosecond transient absorption (ns-TA) and femtosecond transient absorption (fs-TA) [21] study of the photophysics and photochemistry of BQMP-b in aqueous solutions with varying pHs from 7 to 12. Nanosecond time-resolved resonance Raman spectroscopy (Ns-TR^3^) was also applied to detect the vibrational spectra of the intermediate species observed in the photoreaction. In order to figure out the crucial effect of the binaphthol ring on the photochemistry of interest, we also carried out an investigation on compound QMP-b (Scheme 2) with only one naphthol ring in mixed acetonitrile–water (MeCN:H_2_O = 1:1) solutions with different pHs. All of the information gained by time-resolved spectroscopy techniques is then discussed to better understand the data collected for BQMP-b described here. Along with the experimental results, density functional theory (DFT) computations were performed to assist in analyzing the characteristics of the intermediate species. The results indicated that the alkaline condition played a crucial role in the formation of BQMs from BQMP-b, which led to a faster reaction of BQMP-b under strong alkaline aqueous solution conditions. Instead of GSIPT and ESIPT processes, which occur in mild MeCN and water mixed systems, only one process took place in generating the quinone methide species in the corresponding, strongly alkaline aqueous solutions. Collectively, our work here provides new mechanistic insights into the effective formation of BQMs in strong alkaline aqueous solutions. The study about bifunctional quinone methides can also pave the way for future direct time-resolved spectroscopic research on quinone methides alkylating and crosslinking DNA reactions that may occur under various aqueous conditions.

## 2. Results and Discussion

### 2.1. UV-Vis Spectra Study of QMP-b Containing One Naphthol Ring: The Ground State Form Present at pH = 7, 10, and 12 in Mixed Aqueous Solutions

**Figure 1:**
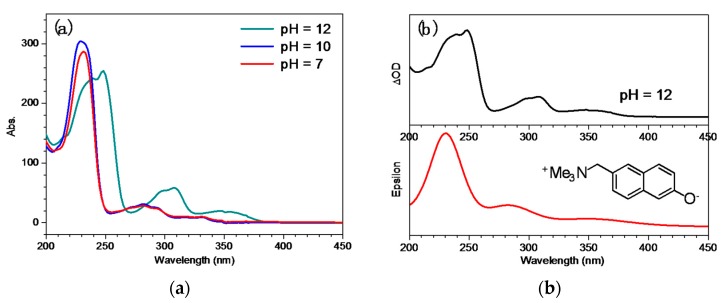
(**a**) QMP-b UV-Vis spectra obtained in mixed aqueous solutions with different pH values. (**b**) UV-Vis spectrum (top) and the computed deprotonated species of QMP-b electronic spectrum (bottom) using the TD-B3LYP/6-311G(d,p) method.

**Scheme 3:**
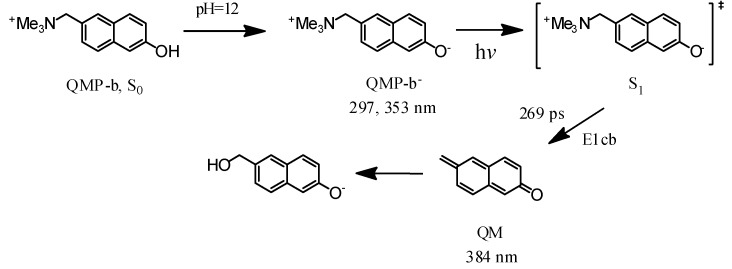
Proposed reaction mechanism for QMP-b in strong alkaline aqueous solution (pH = 12), the (‡) marks the excited state.

Figure 1a presents the UV-Vis spectra of QMP-b obtained in mixed aqueous solutions of pH = 7, 10, and 12. It can be seen that the spectral features in pH = 7 and pH = 10 were almost the same, while they were very different when the pH value reached 12. This phenomenon suggested that the pKa of QMP-b was around 11; the species existing in pH = 7 and pH = 10 was the neutral form of QMP-b, while the strong alkaline condition (pH = 12) led to deprotonation of a hydroxyl group and formation of an anionic species. This process caused significant spectral changes: The deprotonated species absorbed maximally at longer wavelengths than the neutral species, which tailed to 385 nm. Figure 1b shows that the calculated spectra fit very well with the experimental results; The deprotonated species of QMP-b is marked as QMP-b^−^ (Scheme 3). The small differences in the peak positions between the experimental and calculated spectra were likely due to the solvent effect of the fairly simple Polarized Continuum Model (PCM) model used in the calculations, which did not fully account for the actual solvent effect.

### 2.2. Fs-TA Study of QMP-b Containing One Naphthol Ring: Investigating the Early Time Processes after Photoexcitation in Varying pH Mixed Aqueous Solutions

**Figure 2:**
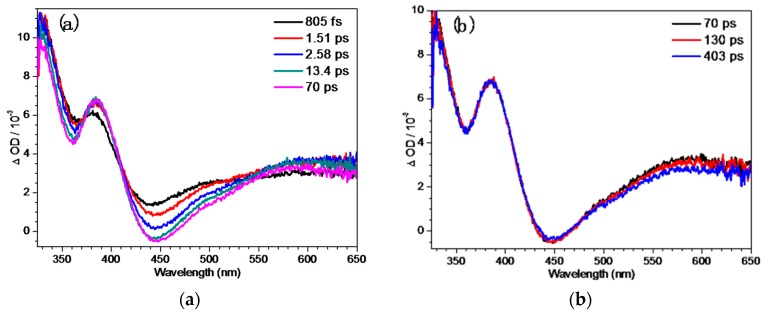

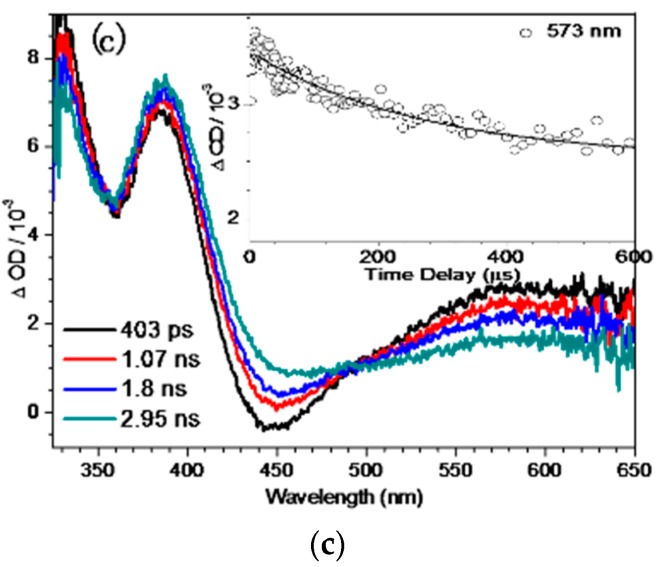
The femtosecond transient absorption (fs-TA) spectra of QMP-b obtained after 266 nm excitation in MeCN:H_2_O (1:1, pH = 12) (**a**) from 1.51 ps to 70 ps, (**b**) from 70 ps to 403 ps, (**c**) from 403 ps to 2.95 ns; inset: kinetics at 573 nm.

In order to study the photoreaction of the binaphthol ring in strong alkaline aqueous solutions, the fs-TA spectra of QMP-b in a pH = 12 mixed aqueous solution were tested and are shown in Figure 2. At earlier times, i.e., from 805 fs to 70 ps (Figure 2a), an absorption band appeared at 385 nm with an apparent stimulated emission band showing up at 440 nm, which could be assigned to the internal conversion (IC) transition from S_n_ to S_1_. In the later times, i.e., from 70 ps to 403 ps, a vibrational cooling process took place. Subsequently, the 573 nm band decreased and the feature at 385 nm intensified gradually, indicating the dynamical conversion between the two different states with formation of a new intermediate. This could probably produce an intermediate QM according to reported work [6,22]. It can be seen in Figure 2c that the kinetics of the decay data at 573 nm was simulated satisfactorily by a single-exponential function (for τ = 269 ps). For comparison, the characterization of QMP-b in pH = 7 and 10 mixed solutions were also conducted (see Figure S1), and no bands assigned to the QM species could be found, indicating that deprotonation had a great impact in the generation of the QM in the photoreaction of QMP-b.

**Figure 3:**
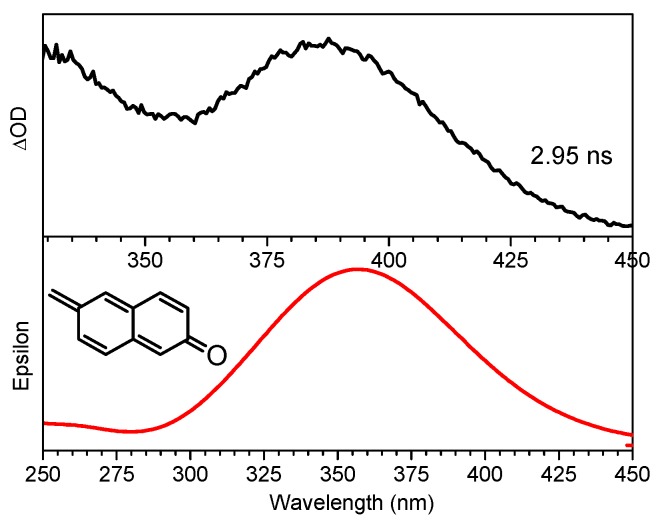
The QMP-b fs-TA spectrum of acquired subsequent to 266 nm photolysis in MeCN:H_2_O (1:1, pH = 12) at 2.95 ns (top) compared to the QM intermediate computed TD-B3LYP/6-311G(d,p) spectrum.

Time-dependent DFT (TD-DFT) calculations have been previously illustrated to be helpful in evaluating and identifying the absorption features of intermediate species [23,24]. Based on the spectral data, a simple mechanism can be deduced that the NMe_3_ group in QMP-b leaves in a process to form the QM species due to an E1cb elimination reaction [25,26]. Therefore, TD-DFT computations were done for the electronic absorption spectra of the intermediate QM to test this hypothesis (see Figure 3). The calculated spectrum of this intermediate showed two broad bands over 250–450 nm, which agreed well with the 2.95 ns experimental spectrum in Figure 3. Hence, this was clear evidence that the TD-DFT spectra could be used to support the generation of a QM intermediate via the singlet excited state of the deprotonated species QMP-b as hypothesized above.

Ns-TA experiments for QMP-b were also performed to determine the later species obtained in the pH = 12 solutions, as shown in Figure S2. The species at 2.95 ns in Figure 3 could also be observed in the ns-TA spectra at early times with two bands at 330 nm and 386 nm. As mentioned above, the species might be attributed to the generation of the QM intermediate (Scheme 3). Afterwards, the two bands decayed slowly with no other peaks appearing over the times studied (Scheme 3). The proposed photoreaction steps derived from the fs-TA and ns-TA results and the DFT calculation of QMP-b in the strong basic aqueous solutions (MeCN:H_2_O = 1:1, pH = 12) are given in Scheme 3.

### 2.3. UV-Vis Spectra Study of BQMP-b Containing Two Naphthol Rings: The Ground State Form Present at pH = 7, 10, and 12 in Mixed Aqueous Solutions

**Figure 4:**
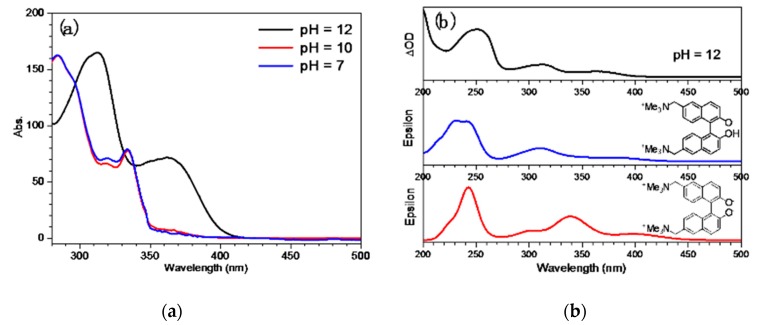
(**a**) UV-Vis spectra of BQMP-b in mixed solution with different pH values. (**b**) UV-Vis spectrum (top) and the BQMP-b calculated TD-B3LYP/6-311G(d,p) electronic spectrum of deprotonated species (middle and bottom).

To determine the BQMP-b ground state structures in different solutions, the BQMP-b UV-Vis spectra were also obtained in MeCN:H_2_O mixed solutions with varying pHs (Figure 4). Obviously, the spectra of BQMP-b observed in pH = 12 solution were quite different to the spectra obtained in pH = 7 and pH = 10 solutions; a similar phenomenon was observed with QMP-b. The deprotonated species in pH = 12 solutions had a broader absorption with a bathochromic shift from 350 nm to 415 nm compared with that in mild aqueous situations. Based on the study of QMP-b, two possible deprotonation forms of BQMP-b were considered (pH = 12, Figure 1b): one where only one hydroxyl group was ionized, losing one proton on O1 and one where two hydroxyl groups in two binaphthol rings were both ionized simultaneously. A comparison of the calculated results with the experimental results showed that the middle spectrum correlated with the experimental data better. Thus, we propose that only one hydroxyl group in BQMP-b is affected in the strong basic aqueous solution at pH = 12 to form the naphtholates BQMP-b^−^.

### 2.4. Fs-TA Study of BQMP-b Containing Two Naphthol Rings: Investigating the Early Time Processes after Photoexcitation in Varying pH Mixed Aqueous Solution

Figures S3 and S4 show the BQMP-b fs-TA spectra acquired in pH = 7 and pH = 10 MeCN:H_2_O = 1:1 aqueous solutions, respectively. Upon 266 nm excitation, two transient absorption features at 351 nm and 440 nm appeared, which were attributed to the IC from S_n_ to S_1_, forming the BQMP-b singlet excited state (Figure S3a). As can be seen in Figure S3b, the initial absorption feature at 351 nm red-shifted a little to 365 nm. Afterwards, two new bands at 389 nm and 496 nm emerged simultaneously. This suggested a new species was produced, which could be assigned to the zwitterionic intermediate 1 (see Scheme 1). At later delay times, the transient absorption bands decayed gradually to form an intermediate 2, which had a broad feature near 365 nm. A similar reaction pathway was also seen in pH = 10 MeCN:H_2_O = 1:1 aqueous solution (Figure S4).

**Figure 5:**
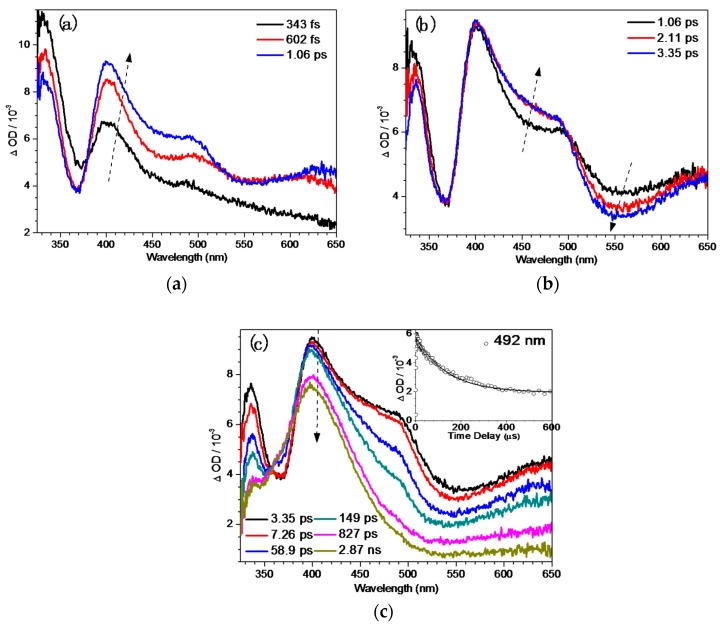
The fs-TA spectra of BQMP-b obtained after 266 nm excitation in MeCN:H_2_O (1:1, pH = 12) (**a**) from 343 fs to 1.06 ps, (**b**) from 1.06 ps to 3.35 ps, (**c**) from 3.35 ps to 2.87 ns; inset: kinetics at 492 nm.

Fs-TA experiments for BQMP-b in pH = 12 demonstrated that the whole process could be partitioned into three time regions to follow a significantly different pathway to BQM formation. In Figure 5, the initial transient species with an absorption peak at 400 nm could be attributed to the S_1_ of the deprotonated BQMP-b^−^ from the S_n_ excited state via the IC process, with a new feature at 492 nm growing in simultaneously. Subsequently, the vibrational cooling process occurred from 1.06 ps to 3.35 ps, which was much faster than that in the QMP-b system due to the additional binaphthol ring, which could speed up the rotation of the structure in BQMP-b (Figure 5b). At later times, one intermediate with a predominant 400 nm band was still around at 2.9 ns (Figure 5c). The inset in Figure 5c exhibits the kinetics of the singlet decay at 492 nm of BQMP-b^−^, which can be fitted to a time constant of 136 ps using a single-exponential function. This process could potentially be the formation of BQM species. The kinetics revealed that only one process took place in the conversion from the S_1_ of the deprotonated BQMP-b^−^ to produce BQM.

**Figure 6:**
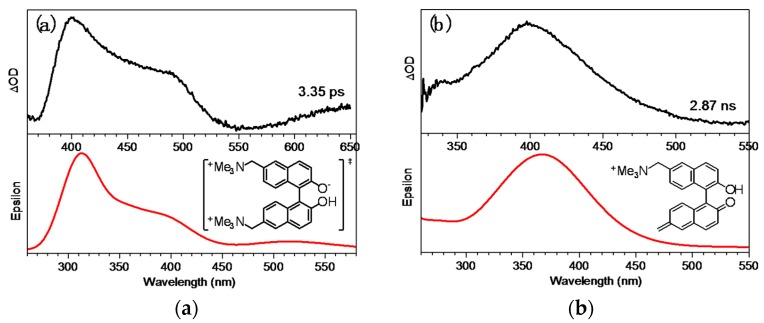
(**a**) The fs-TA spectrum of BQMP-b obtained after 266 nm excitation in MeCN:H_2_O (1:1, pH = 12) at 3.35 ps (top) and the computed spectrum of the singlet excited state of BQMP-b^−^. (**b**) The fs-TA spectra of BQMP-b obtained after 266 nm excitation in MeCN:H_2_O (1:1, pH = 12) at 2.87 ns (top) and the computed spectrum of intermediate BQM from TD-B3LYP/6-311G(d,p) calculations.

To make a more detailed analysis, a comparison of the TD-DFT-calculated spectra for the BQMP-b^−^ S_1_ state and BQM species with the corresponding fs-TA experimental spectra are displayed in Figure 6. The computed S_1_ spectral result for BQMP-b^−^ showed two main features in the 200–600 nm region, and the spectrum for the BQM species only had one absorption band on this wavelength scale. In Figure 6, the DFT-calculated Raman spectra are both in reasonable agreement with the fs-TA spectra at 3.35 ps in Figure 6a and at 2.87 ns in Figure 6b. Optimized geometry parameters for BQMP-b^−^ in the S_0_ and S_1_ states were also investigated (see Figure S5). These results showed that the S_1_ of BQMP-b^−^ had a shorter bond between C2-O1 (1.26 Å, right) in the binaphthol ring group compared to that of BQMP-b^−^ in the ground state (1.29 Å, left), which further suggested that this C–O bond had high tendencies to form the C=O bond. According to the mechanism of an E1cb elimination reaction, the lone electron on the O1 anion quickly moved to the neighboring carbon atom C2 in the binaphthol ring to form a carbanion and then transferred rapidly to the C3 position due to the conjugated structure in the binaphthol ring group (Scheme 2), expelling the leaving group of NMe_3_ and forming the C=C double bond simultaneously [27,28]. It is reasonable that the reactive efficiency for generating QM in a strong basic aqueous solution could be much higher than that produced by a GSIPT process in mild MeCN:H_2_O solutions. This can also be deduced by the data obtained from ns-TR^3^ data shown in the following section.

### 2.5. Ns-TR^3^ Study of BQMP-b Containing Two Naphthol Rings: Characterizing the Structure and Properties of Key Intermediates

Figure S6 displays TR^3^ spectra acquired at different delay times after photolysis of BQMP-b in a MeCN:H_2_O (1:1) mixed solvent with pH = 12. The TR^3^ spectra had six Raman bands at 1622 cm^−1^, 1580 cm^−1^, 1563 cm^−1^, 1519 cm^−1^, 1233 cm^−1^, and 1139 cm^−1^. According to the investigation of fs-TA for BQMP-b, the transient species observed in pH = 12 solution was likely BQM. Figure S7 presents a comparison ns-TR^3^ spectra acquired at 1 µs in pH = 7 and pH = 12 mixed solutions. The two spectra were almost identical except for the relative intensities, indicating that the species produced in pH = 7 could also been observed in pH = 12. Close inspection indicated that this intermediate had a larger intensity in the pH = 12 solution than seen in pH = 7 solution. Photolysis of BQMP-b in a MeCN:H_2_O (1:1, pH = 7) has previously been observed to produce BQM intermediate on the ns to μs time regions [4], which can provide evidence that the intermediate seen in Figure S6 could be assigned to the BQM intermediate. This intermediate was produced much faster in pH = 12 mixed aqueous conditions.

**Figure 7:**
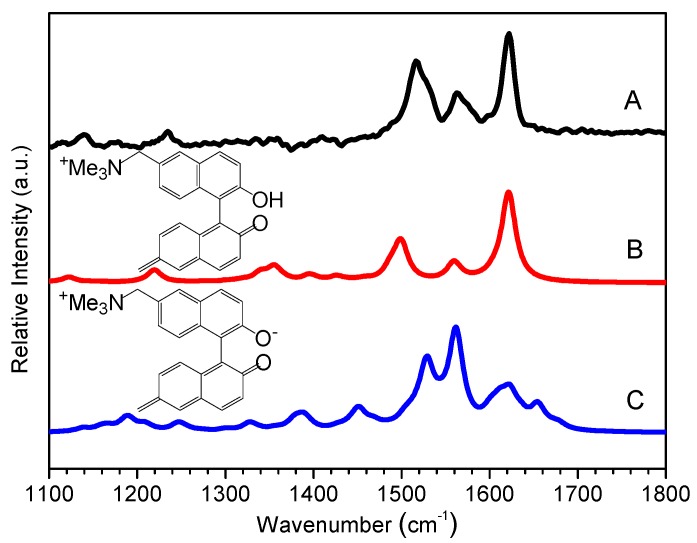
(**A**)Experimental BQMP-b TR^3^ spectrum (at 1μs) seen in MeCN:H_2_O (1:1, pH = 12) compared to a density functional theory (DFT)-calculated Raman spectrum of (**B**) BQM and (**C**) BQM^−^ species.

**Scheme 4:**
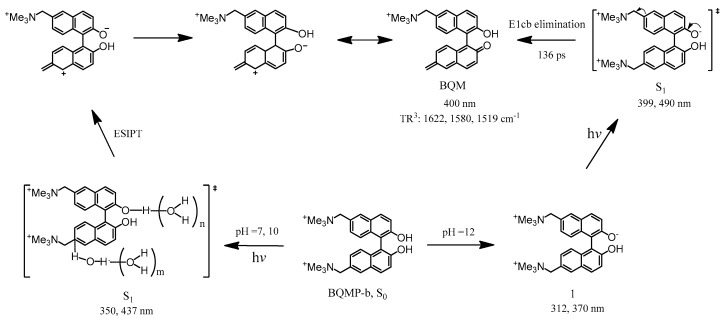
Proposed reaction mechanism for BQMP-b based on the results from the time-resolved spectroscopy experiments, the (‡) marks the excited state.

To confirm the assignment of BQM species in ns-TR^3^, DFT computations were also carried out to estimate the normal Raman spectra of BQM and BQM^−^. Figure 7 compares the experimental TR^3^ spectra observed at 1 μs to the calculated normal Raman spectrum of BQM and BQM^−^. Most calculated Raman bands for BQM, e.g. 1622 cm^−1^, 1563 cm^−1^, 1502 cm^−1^, and 1225 cm^−1^ bands were in excellent agreement with the experimental ns-TR^3^ spectra, while no analogous agreement was found between the TR^3^ spectra and the computed Raman spectrum of BQM^−^, which showed that the intermediate found in the ns time region might be attributed to BQM. Thus, a detailed assignment of the vibrational modes could be deduced due to the good consistency between the experimental and simulated BQM Raman bands. The 1622 cm^−1^ band was assigned to the stretching vibrational mode of the quinone methide ring, the 1563 cm^−1^ band was attributed to the naphthyl ring stretching vibration, and the 1233 cm^−1^ band corresponded to the C–H in-plane rocking mode of the rings. According to our previous work, the addition of water molecules into the system may have an effect on the computed results due to the formation of hydrogen bonding between carbonyl and the hydroxyl moieties with water molecules [17]. Figure S8 exhibits the results without adding water molecules and adding one or two water molecules into the BQM^−^ system. It can be seen that no calculated curves appear to agree well with the experimental spectrum, suggesting the possible very low concentration or nonexistence of the BQM^−^ species. All the results based on these fs-TA, ns-TR^3^, and DFT data, as well as on previous related literature, provide strong evidence for the quicker formation of the BQM species in pH = 12 aqueous solution. A proposed mechanism for the photoreaction of BQMP-b in a MeCN:H_2_O (1:1, pH = 12) system is outlined in Scheme 4.

## 3. Materials and Methods

### 3.1. Materials

The BQMP-b compound was prepared using methods previously noted in the literature [4]. Distilled water and spectroscopic grade acetonitrile (MeCN) were utilized to prepare samples using sodium hydroxide (NaOH) employed as required to vary the pH values of the solvent in the experiments. Specifically, the MeCN:H_2_O = 1:1 mixed solution was adjusted to pH = 7, 10, or 12 by adding NaOH. Then, a known amount of BQMP-b was added into the mixed solvent for the experiments. The volume ratios were the same as the mixed solution ratios.

### 3.2. Ns-TA and fs-TA Experiments

The experiments here used the same experimental setups and methods detailed in previous work [24,29].

### 3.3. Ns-TR^3^ Experiments

The ns-TR^3^ operations were discussed in previous work [30,31].

### 3.4. DFT-Calculations

The DFT calculations were performed using the B3LYP method with a 6-311G(d,p) basis set. The predicted spectra were derived by a Lorentzian function, with a 10 cm^−1^ bandwidth applied to the Raman vibrational frequencies and a 2500 cm^−1^ bandwidth for the UV-Vis spectra. The number of states computed was 40, and a scaling factor of 0.975 was applied to the vibrational frequencies. Solvent effects were considered a using PCM model. All the calculations were performed employing the Gaussian 09 program, more details of which are described in a previous related study [32]. Note that all TD-DFT calculations used the vertical approximation, an approximation that might come with significant errors [33].

## 4. Conclusions

The study detailed here has given a helpful elucidation of the electrophilic properties of BQMP-b in neutral and basic aqueous solutions. The pH of the solution obviously plays a key factor in determining the photogeneration of the BQMs and also the selectivity of their alkylation reactions with various substrates. The key findings of our work here are as follows: (1) In neutral aqueous condition, BQMP-b experienced ESIPT and GSIPT processes in the photoreaction. However, instead of a proton transfer, the E1cb elimination reaction took place under strong basic aqueous conditions, which is a new finding for the photochemistry of BQMP-b. (2) The most attractive finding is that the reactive BQM species that can be generated from BQMP-b in neutral aqueous condition was also detected in a strong alkaline aqueous solution but with higher efficiency in the strong alkaline aqueous condition. This suggests that there may be room for improvement in the efficiency of QM generation at selected pH aqueous solutions by judiciously varying the properties of the BQMs. Due to the potential of BQM for cancer therapy, all the clear spectroscopic and theoretical characterizations of the intermediates involved in the processes for the BQM generation help to better elucidate important benchmarks that may be used in future studies into photoinitiated BQM reactions toward DNA in biological systems.

## Supplementary Materials

The following are available online. Figure S1: The fs-TA spectra of QMP-b obtained after 266 nm excitation in MeCN:H_2_O (a) from 1.52 ps to 2.91 ns (1:1, pH = 7), (b) from 2.32 ps to 2.86 ns (1:1, pH = 10). Figure S2: Ns-TA spectra of QMP-b after 266 nm photolysis in MeCN:H_2_O (1:1, pH = 12) mixed solution. Figure S3: The fs-TA spectra of BQMP-b obtained after 266 nm excitation in MeCN:H_2_O (1:1, pH = 7) (a) from 224 fs to 828 fs, (b) from 828 fs to 20.8 ps, (c) from 20.8 ps to 2.83 ns. Figure S4: The fs-TA spectra of BQMP-b obtained after 266 nm excitation in MeCN:H_2_O (1:1, pH = 10) (a) from 159 fs to 742 fs, (b) from 742 fs to 34.8 ps, (c) from 34.8 ps to 2.83 ns. Figure S5: Schematic depiction of the optimized structures of the ground state of BQMP-b^−^ (left) and singlet excited state of BQMP-b^−^ (right) obtained from B3LYP/6-311G(d,p) DFT calculations. Selected bond lengths (in Å) are labeled in the structures. Figure S6: Shown are the 416 nm probe ns-TR^3^ spectra obtained after 266 nm photolysis of BQMP-b in MeCN:H_2_O (1:1) mixed solvent with pH = 12. Figure S7: Comparison of the ns-TR^3^ spectra obtained at 10 ns in pH = 7 and pH = 12 mixed solutions. Figure S8: Experimental TR^3^ spectrum (at 1μs) of BQMP-b observed in MeCN:H_2_O (1:1, pH = 12, 266 nm pump, 416 nm probe) compared to DFT computed Raman spectrum of BQM^−^ species.
