# Supplementary material for: Time-Resolved Spectroscopic and Density Functional Theory Investigation of the Photogeneration of a Bifunctional Quinone Methide in Neutral and Basic Aqueous Solutions
Source: Molecules. 2018 Nov 27;23(12):3102. doi: 10.3390/molecules23123102 (PMC6321171; doi:10.3390/molecules23123102)
Supplement: Supplementary file 1 [file molecules-23-03102-s001.zip › Molecules-Special-Issue-KCG-editor-BQM-SI-Oct-7-2018.pdf]

Supplementary Materials for

Time-Resolved Spectroscopic and Density Functional Theory Study of the Photogeneration of a Bifunctional Quinone Methide in Neutral and Basic Aqueous Solutions

Zhiping Yan [1], Lili Du [1,*], Xin Lan [1], Yuanchun Li [1], Wenchao Wang [1] and David Lee Phillips [1,*]

[1] Department of Chemistry, The University of Hong Kong, Hong Kong S.A.R., China; mcayzp@gmail.com (Z.Y.); xinlan@connect.hku.hk (X.L.); fionalyc@connect.hku.hk (Y.L.); wenchao0909@126.com (W.W.)

* Correspondence: justailleen@gmail.com (L.D.); phillips@hku.hk (D.L.P.); Tel.: +852-6761-4757 (L.D.); +852-2859-2160 (D.L.P.)

Figure S1. The fs-TA spectra of QMP-b obtained after 266 nm excitation in MeCN:H_2O (a) from 1.52 ps to 2.91 ns (1:1, pH = 7), (b) from 2.32 ps to 2.86 ns (1:1, pH = 10)..S**Error! Bookmark not defined.**

Figure S2. Ns-TA spectra of QMP-b after 266 nm photolysis in MeCN:H_2O (1:1, pH = 12) mixed solutions..S2

Figure S3. The fs-TA spectra of BQMP-b obtained after 266 nm excitation in MeCN:H_2O (1:1, pH = 7) (a) from 224 fs to 828 fs, (b) from 828 fs to 20.8 ps, (c) from 20.8 ps to 2.83 ns...S2

Figure S4. The fs-TA spectra of BQMP-b obtained after 266 nm excitation in MeCN:H_2O (1:1, pH = 10) (a) from 159 fs to 742 fs, (b) from 742 fs to 34.8 ps, (c) from 34.8 ps to 2.83 ns..S**Error! Bookmark not defined.**

Figure S5. Schematic depiction of the optimized structures of the ground state of BQMP-b$^-$ (left) and singlet excited state of BQMP-b$^-$ (right) obtained from B3LYP/6-311G** DFT calculations. Selected bond lengths (in Å) are labeled in the structures..S**Error! Bookmark not defined.**

Figure S6. Shown are the 416 nm probe ns-TR^3 spectra obtained after 266 nm photolysis of BQMP-b in MeCN:H_2O (1:1) mixed solvent with pH = 12. ....S**Error! Bookmark not defined.**

Figure S7. Comparison of the ns-TR^3 spectra of BQMP-b obtained at 1 μs in pH = 7 and pH = 12 mixed solutions..S4

Figure S8. Experimental TR^3 spectrum (at 1us) of BQMP-b observed in MeCN:H_2O (1:1, pH = 12, 266 nm pump, 416 nm probe) compared to DFT computed Raman spectrum of BQM$^-$ species. S4

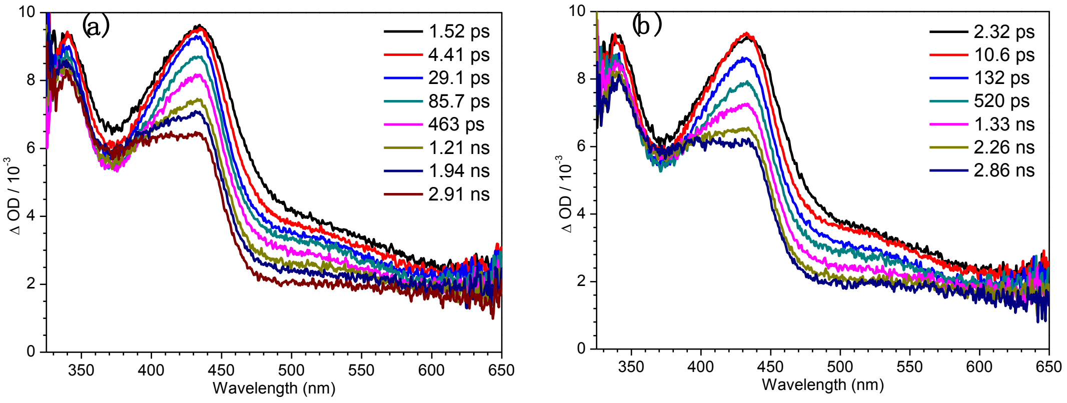

Figure S1. The fs-TA spectra of QMP-b obtained after 266 nm excitation in MeCN:H_2O (a) from 1.52 ps to 2.91 ns (1:1, pH = 7), (b) from 2.32 ps to 2.86 ns (1:1, pH = 10).

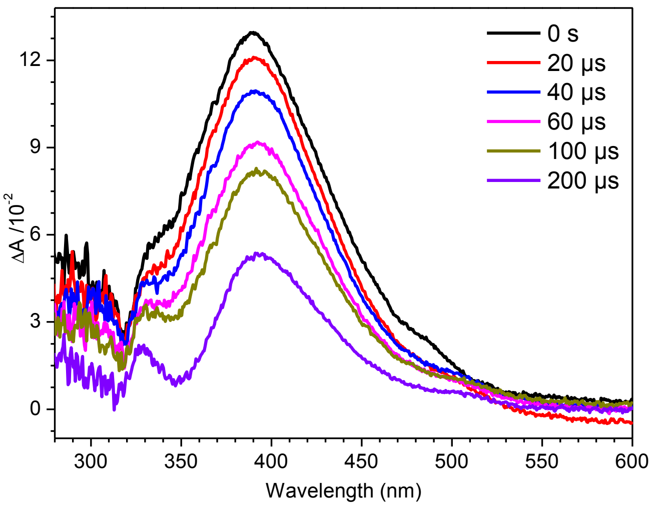

Figure S2. Ns-TA spectra of QMP-b after 266 nm photolysis in MeCN:H_2O (1:1, pH = 12) mixed aqueous solutions.

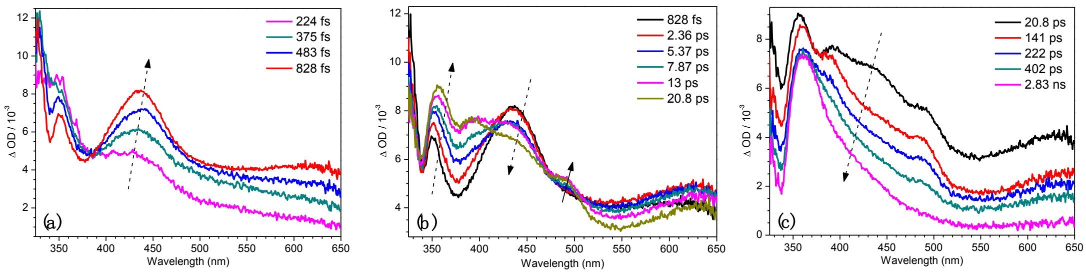

Figure S3. The fs-TA spectra of BQMP-b obtained after 266 nm excitation in MeCN:H_2O (1:1, pH = 7) (a) from 224 fs to 828 fs, (b) from 828 fs to 20.8 ps, (c) from 20.8 ps to 2.83 ns.

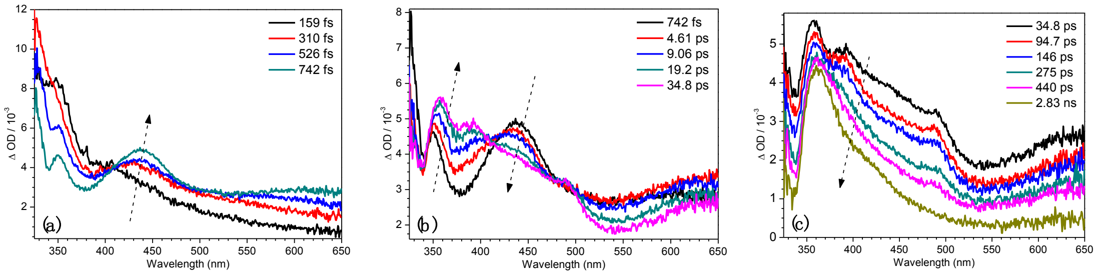

Figure S4. The fs-TA spectra of BQMP-b obtained after 266 nm excitation in MeCN:H_2O (1:1, pH = 10) (a) from 159 fs to 742 fs, (b) from 742 fs to 34.8 ps, (c) from 34.8 ps to 2.83 ns.

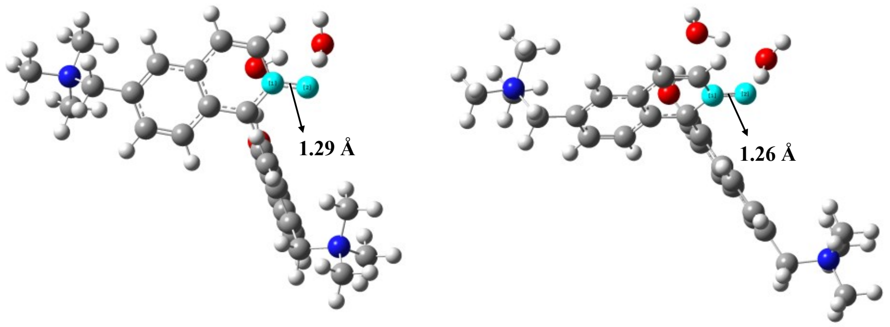

Figure S5. Schematic depiction of the optimized structures of the ground state of BQMP-b$^-$ (left) and singlet excited state of BQMP-b$^-$ (right) obtained from B3LYP/6-311G** DFT calculations. Selected bond lengths (in Å) are labeled in the structures.

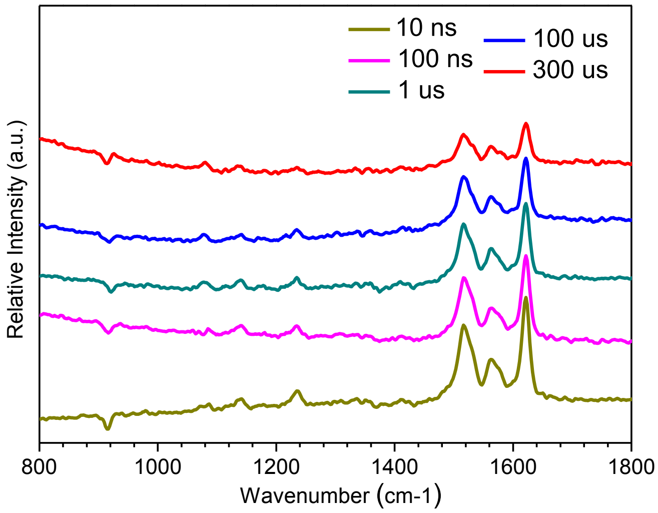

Figure S6. Shown are the 416 nm probe ns-TR[3] spectra obtained after 266 nm photolysis of BQMP-b in MeCN:H_2O (1:1) mixed solvent with pH = 12.

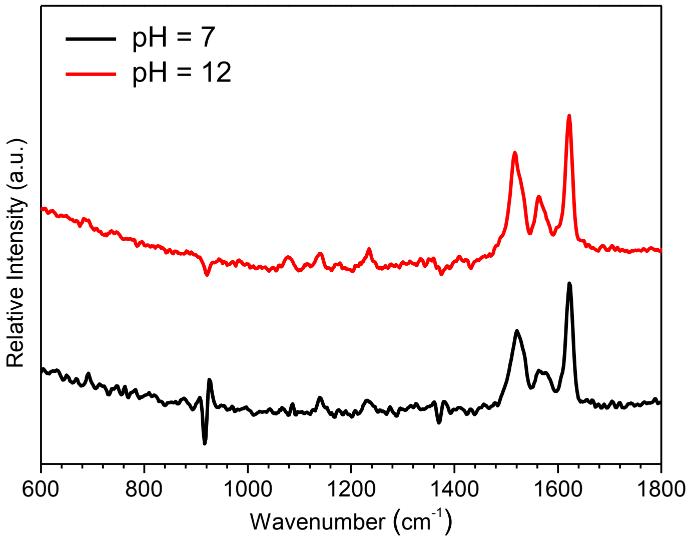

Figure S7. Comparison of the ns-TR[3] spectra of BQMP-b obtained at 1 μs in pH = 7 and pH = 12 mixed aqueous solutions.

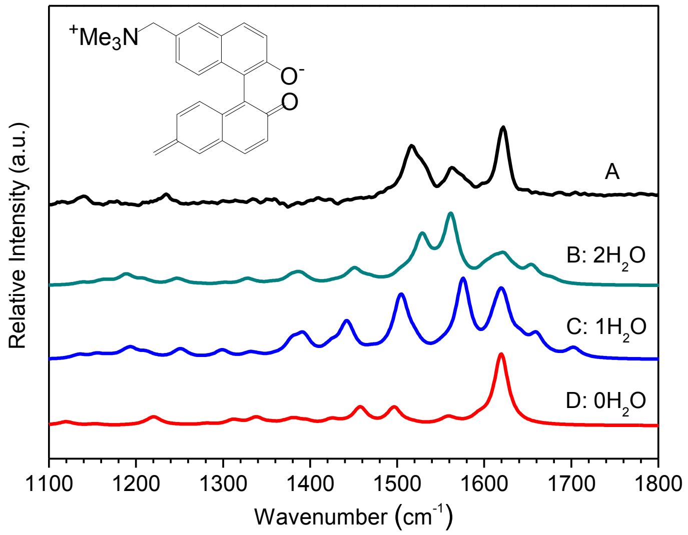

Figure S8. Experimental TR[3] spectrum (at 1us) of BQMP-b observed in MeCN:H_2O (1:1, pH = 12, 266 nm pump, 416 nm probe) compared to DFT computed Raman spectrum of the BQM^- species.
